# Inhibition of *Histone Deacetylase 3* Causes Replication Stress in Cutaneous T Cell Lymphoma

**DOI:** 10.1371/journal.pone.0068915

**Published:** 2013-07-22

**Authors:** Christina E. Wells, Srividya Bhaskara, Kristy R. Stengel, Yue Zhao, Bianca Sirbu, Benjamin Chagot, David Cortez, Dineo Khabele, Walter J. Chazin, Andrew Cooper, Vincent Jacques, James Rusche, Christine M. Eischen, Laura Y. McGirt, Scott W. Hiebert

**Affiliations:** 1 Department of Biochemistry, Vanderbilt University School of Medicine, Nashville, Tennessee, United States of America; 2 Departments of Radiation Oncology and Oncological Sciences, Huntsman Cancer Institute, University of Utah School of Medicine, Salt Lake City, Utah, United States of America; 3 Laboratoire AREMS UMR7214 UL-CNRS, Université de Lorraine, Vandoeuvre-Lés-Nancy, France; 4 Vanderbilt-Ingram Cancer Center, Vanderbilt University School of Medicine, Nashville, Tennessee, United States of America; 5 Department of Cancer Biology, Vanderbilt University Medical Center, Nashville, Tennessee, United States of America; 6 Department of Obstetrics and Gynecology, Division of Gynecologic Oncology, Vanderbilt University Medical Center, Nashville, Tennessee, United States of America; 7 Repligen Corporation, Waltham, Massachusetts, United States of America; 8 Department of Pathology, Microbiology and Immunology, Vanderbilt University Medical Center, Nashville, Tennessee, United States of America; 9 Department of Medicine, Division of Dermatology, Vanderbilt University Medical Center, Nashville, Tennessee, United States of America; St. Georges University of London, United Kingdom

## Abstract

Given the fundamental roles of histone deacetylases (HDACs) in the regulation of DNA repair, replication, transcription and chromatin structure, it is fitting that therapies targeting HDAC activities are now being explored as anti-cancer agents. In fact, two histone deacetylase inhibitors (HDIs), SAHA and Depsipeptide, are FDA approved for single-agent treatment of refractory cutaneous T cell lymphoma (CTCL). An important target of these HDIs, histone deacetylase 3 (HDAC3), regulates processes such as DNA repair, metabolism, and tumorigenesis through the regulation of chromatin structure and gene expression. Here we show that HDAC3 inhibition using a first in class selective inhibitor, RGFP966, resulted in decreased cell growth in CTCL cell lines due to increased apoptosis that was associated with DNA damage and impaired S phase progression. Through isolation of proteins on nascent DNA (iPOND), we found that HDAC3 was associated with chromatin and is present at and around DNA replication forks. DNA fiber labeling analysis showed that inhibition of HDAC3 resulted in a significant reduction in DNA replication fork velocity within the first hour of drug treatment. These results suggest that selective inhibition of HDAC3 could be useful in treatment of CTCL by disrupting DNA replication of the rapidly cycling tumor cells, ultimately leading to cell death.

## Introduction

Cutaneous T cell lymphoma (CTCL) is a heterogeneous group of non-Hodgkin’s lymphoma that is characterized by accumulation of malignant T cells in the skin [1]–[3]. The most common subtypes of CTCL are mycosis fungoides, Sézary Syndrome, and the CD30^+^ lymphoproliferative disorders, comprising 95% of CTCL [2]–[5]. Histone deacetylase (HDAC) inhibitors have become an important treatment option for CTCL that progresses to the more aggressive stages of disease. Histone deacetylases are likely to serve as valuable therapeutic targets as they contribute to genomic stability and cell cycle control through their fundamental roles in cell proliferation including the regulation of DNA repair, replication, transcription, and chromatin structure. In fact, due to their success in the treatment of CTCL, HDACs are now being explored as therapeutic targets for multiple cancers [6]–[9].

Two histone deacetylase inhibitors (HDIs), SAHA (Vorinostat) and Depsipeptide (Romidepsin), are FDA approved for the treatment of refractory CTCL [1], [3], [10]–[12]. Both of these compounds inhibit multiple HDACs with SAHA inhibiting class I and II HDACs while Depsipeptide inhibits the class I HDACs and HDAC6 [10], [11], [13]. However, since these HDIs inhibit multiple HDACs, they may be inhibiting targets that are not integral to CTCL survival and progression, thereby causing unnecessary side effects. Treatment with SAHA or Depsipeptide is less toxic than standard chemotherapy but can be associated with negative impacts on quality of life [3], [12], [13]. Adverse effects of SAHA and Depsipeptide include nausea, fatigue, gastrointestinal and cardiac toxicity, and hematologic impairment [3], [12], [13]. Additionally, the roles of HDACs in tumorigenesis and the mechanisms by which HDAC inhibition is effective against cancer remain unclear. Therefore, selective inhibition of HDACs may decrease side effects by inhibiting only one or two HDACs at a time and allow for further elucidation of the roles of individual HDACs in cancer.

An important target of these HDIs is histone deacetylase 3, or HDAC3. HDAC3 (a class I HDAC) is involved in the regulation of chromatin structure and gene expression, which controls DNA repair, metabolism, and even tumorigenesis [14]–[18]. While HDACs are often thought of exclusively as transcriptional repressors, mouse embryonic fibroblasts (MEFs) lacking HDAC3 displayed S phase dependent DNA damage accumulation, deregulation of transcription, and apoptosis [17]. Due to this role in DNA damage, selective HDAC3 inhibition could potentially target the rapidly proliferating tumor cells while not harming the surrounding quiescent, non-malignant cells [19]–[24].

HDACs are classified based on sequence conservation. The class I HDACs (HDACs 1, 2, 3, and 8) are homologous to yeast RPD3 while the class II HDACs are more similar to the yeast Hda1 enzyme [25]–[28]. HDACs 1 and 2 share 82% identity while these HDACs share 53% and 52% identity, respectively, with HDAC3 [29]–[31]. The class I HDACs also contain a highly conserved central catalytic domain [30], [31] that is 58% identical between HDAC1 and HDAC3. Given the high level of homology between the class I HDACs, it is understandable why a selective inhibitor would be difficult to identify. However, a new class of inhibitors, N-(*o*-aminophenyl) carboxamides, can show 10-fold or higher selectivity for HDAC3, over HDACs 1 and 2 [[32] and Vincent Jacques, Repligen, unpublished data]. This family of inhibitors includes RGFP966 [32]–[35], which has an IC_50_ of 0.08 µM in *in vitro* substrate assays and inhibition of other HDACs by RGFP966 was not seen at concentrations up to 15 µM [32]. Therefore, we set out to determine the effects of selective HDAC3 inhibition using RGFP966 on cancer cell growth.

Here we treated CTCL cell lines with a selective HDAC3 inhibitor and found that these cells exhibited sensitivity to selective HDAC3 inhibition as demonstrated by decreased cell growth and increased apoptosis. We also found that these cells had increased DNA damage upon HDAC3 inhibition and did not progress normally through the cell cycle due to impaired S phase progression. Consistently, DNA fiber labeling assays demonstrated that inhibition of HDAC3 caused a 50% reduction in DNA replication fork velocity. Through isolation of proteins on nascent DNA (iPOND), we determined that Hdac3 is associated with chromatin and present at and around DNA replication forks. Thus, HDAC3 inhibition caused replication stress in CTCL cells, and selective inhibition of HDAC3 through novel inhibitors may be useful in the treatment of CTCL.

## Materials and Methods

### Ethics Statement

Mouse studies were performed under an animal protocol approved by the Vanderbilt Institutional Animal Care and Use Committee, Nashville, TN.

### Cell Culture

HH (CD30^+^ lymphoproliferative disorder) cells (ATCC) were cultured in RPMI supplemented with 10% heat inactivated fetal bovine serum (FBS), 50 U/ml penicillin, 50 µg/ml streptomycin, and 2 mM _L_-glutamine. Hut78 (Sézary Syndrome) cells (ATCC) were cultured in Iscove’s Modified Dulbecco’s Medium (IMDM) supplemented with 20% heat inactivated FBS, 50 U/ml penicillin, 50 µg/ml streptomycin, and 4 mM _L_-glutamine. Cells were maintained between 2×10^5^–1×10^6^ cells/mL.

### Antibodies

The following antibodies were purchased from Abcam: Histone H4 [EP10000Y] (acetyl K5) (ab51997), Histone H3 (acetyl K27) (ab4729), HDAC 1 (ab19845), HDAC 2 [Y461] (ab32117), HDAC 3 (ab16047) and Histone H2B (ab1790). Histone H3 [96C10] (3638S) and Histone H4 [L64C1] (2935S) were used as loading controls and purchased from Cell Signaling. Anti-acetyl histone H3 (or H3K9K14ac) (06–599) and Anti-phospho-Histone H2A.X (Ser 139) clone JBW301 (05–636) were purchased from Millipore. Histone H3 (acetyl K56) (2134-1) was purchased from Epitomics, and anti-actin (A2066) was purchased from Sigma Aldrich. PCNA [FL261] was purchased from Santa Cruz (SC7907).

### Histone Deacetylase Inhibitors (HDIs) and CTCL Therapeutic Drugs

Depsipeptide (aka Romidepsin, FK228, Depsi) was kindly provided by Celgene. The HDIs RGFP233, RGFP136, and RGFP966 were synthesized and kindly given to us by Repligen Corporation. These compounds are analogs of previously published compounds [34] but have different HDAC inhibition selectivity [32]–[35]. In purified enzyme assays, RGFP966, 233, and 136 had the following HDAC inhibition IC_50_ values for HDAC1, HDAC2, and HDAC3: RGFP966: >15, >15, 0.08 µM; RGFP233∶0.034, 0.059, 3.33 µM; and RGFP136∶5.2, 3.0, 0.4 µM. Bexarotene (SML0282), Methotrexate (M8407), and ATRA (R2625) were purchased from Sigma Aldrich.

### Protein Preparation and Western Blot Analysis

For preparation of whole cell protein lysates, cell pellets were washed with PBS and then sonicated in radioimmunoprecipitation assay (RIPA) buffer containing protease inhibitors (Roche) and phosphatase inhibitors (Roche). For preparation of liver lysates, livers were minced in RIPA buffer with protease inhibitors with a razor blade and then homogenized using a dounce homogenizer. Samples were sonicated and then cleared by centrifugation. Then samples were diluted 1∶2 in Laemmli’s sample buffer (Bio-Rad) and subjected to 13% sodium dodecyl sulfate-polyacrylamide gel electrophoresis. Western blot analyses were performed using primary antibodies listed above and for histone modification or γH2ax westerns, fluorophore conjugated secondary antibodies and the Odyssey system (LiCor) were used. For the iPOND experiment, a HRP secondary antibody and Western Lightning Plus enhanced chemiluminescence substrate (PerkinElmer, NEL103001EA) was used.

For protein separation, soluble chromatin obtained from Hela cells was fractionated using a Superose 6 10/300 GL (GE Healthcare) gel filtration column. Fractions (0.5 ml) were collected, concentrated using trichloroacetic acid precipitation, and analyzed by western blotting using the antibodies indicated in the figure legends. Molecular weight standards were added to the sample as controls. Their elution fractions are indicated at top of the figure.

### Growth Curves

Alamar blue was purchased from Invitrogen (DAL1100). Cells were counted and split into T25 (Corning) flasks at 2×10^5^ cells/mL. Cells were then treated with DMSO, or HDIs once at hour 0. 100 µl aliquots were taken in triplicate from each flask at 0 hr, 24 hrs, 48 hrs, and 72 hrs after treatment, distributed into a flat bottom 96-well plate, and 10 µl of alamar blue added to each well. After a 4 hr incubation, fluorescence was measured using the Biotek Synergy MX Microplate Reader. For the dual treatment curves, the same protocol was followed except ATRA was re-administered at 48 hrs after the initial treatment.

### Annexin V Staining

Annexin V analysis of HH and Hut78 cells was performed using annexin V-fluorescein isothiocyanate (annexin V-FITC) apoptosis detection kit I (BD Pharmingen - 556547) per the manufacturer’s instructions. Briefly, cells were treated with DMSO, Depsi, or HDIs for 24 hrs, pelleted, washed with PBS, and counted. Cells were then resuspended in annexin V binding buffer, labeled with annexin V-FITC and propidium iodide (PI), and then analyzed by flow cytometry using the 5-laser BD LSRII instrument in the Vanderbilt Flow Cytometry Core. Here propidium iodide (PI) is used as a vital dye.

### BrdU Staining

Cell cycle status was analyzed using the FITC Mouse Anti-BrdU set (BD Pharmingen-556028). Cells were treated with DMSO, Depsi, or HDIs for 24 hrs and then BrdU (20 µM final concentration) was added to each flask one and a half hours before harvesting. The cells were then pelleted, washed with PBS, and counted. 1×10^6^ cells per sample were pelleted, resuspended in 200 µl cold PBS and 5 mls of cold 100% ethanol, covered with foil, and stored at 4°C overnight. The next day cells were pelleted, resuspended in 1 mL 2N HCL supplemented with 0.5 mg/mL pepsin, and then incubated for exactly 30 mins at 37°C. Samples were then neutralized with 3 mL 0.1M Sodium Tetraborate (pH 8.5) and pelleted for 7 mins. Then samples were washed 1× with 1 mL of PBS +0.5% BSA, pelleted, washed 1× with PBS +0.5% BSA +0.5% Tween 20, and pelleted again. Samples were then resuspended in FITC-Conjugated anti-BrdU and incubated for 45 mins at room temperature in the dark. Samples were washed one more with PBS +0.5% BSA +0.5% Tween 20 and resuspended in 400 µL of PBS. Propidium iodide and RNase A were added to each sample and then analyzed by flow cytometry using the 5-laser BD LSRII instrument in the Vanderbilt Flow Cytometry Core.

### iPOND

Analysis of proteins associated with DNA replication forks was performed using the iPOND (isolated proteins on nascent DNA) method described previously [36]. Briefly, Hut78 cells were pulsed with EdU for 15 mins followed by either no thymidine chase or a 60 minute thymidine chase. The protein-DNA complexes were then crosslinked with 1% (wt/vol) formaldehyde, nascent DNA was conjugated to biotin using click chemistry, and then protein-DNA complexes were purified using Streptavidin beads. The eluted proteins were then analyzed using western blot analysis. A no click reaction sample (No Clk) that did not include biotin azide was used as a negative control. 0.1% input samples were included for positive controls of each protein analyzed. PCNA served as a positive control for a replication fork associated protein and H2B served as a loading control and positive control for a chromatin associated protein.

### DNA Fiber Labeling

DNA fiber labeling analysis was used to assess DNA replication fork progression [37] in Hut78 cells treated with DMSO, 10 nM Depsipeptide or 10 µM 966. For experiments where DMSO or HDIs were added prior to labeling, DMSO or HDIs were added 5 mins or 4 hrs prior to the addition of IdU (green). Following a 20 min IdU pulse (20 µM final concentration), cells were washed and drug re-administered along with 100 µM CldU for 20 mins. Cells were then washed with equilibrated HBSS, resuspended in cold PBS at 1×10^6^ cells/ml, and mixed with non-labeled cells for better spreading results (20 µL labeled cells +60 µL non-labeled cells). 2 µL of cell suspension and 10 µL of spreading buffer (0.5% SDS, 200 mM Tris-HCl ph 7.4, 50 mM EDTA) was added to each slide, let sit for 6 mins at RT and then tilted to 15 degrees to allow the DNA to run slowly down the slide. 5 slides were made for each sample. Slides were then air dried for at least 40 mins, fixed in 3∶1 methanol:acetic acid for 2 mins, air dried again for 20 mins, and then stored at 4°C overnight.

The next day, slides were submerged in 2.5M HCl for 30 mins, rinsed 3× in PBS and then incubated in 10% goat serum/0.1%Triton in PBS for 1 hr. Then slides were incubated in the dark for 1 hr in rat monoclonal anti-CldU (Accurate Chemical OBT0030G) and mouse anti-IdU (Becton Dickinson 347580) diluted 1/100 in 10% goat serum/0.1% Triton in PBS. Slides were then rinsed 3× in PBS and incubated 30 min with secondary antibodies (Invitrogen Alexa Fluor 568 goat anti-rat-IgG A-11077 and Alexa Fluor 488 goat anti-mouse-IgG A-11029) in 10% goat serum/0.1% Triton in PBS in the dark. Slides were then rinsed 3× in PBS, air dried in the dark, mounted with 110 µL of Prolong Gold with no Dapi (Invitrogen P36930) using whole slide coverslips, let dry overnight at RT and then stored at 4°C. Samples were imaged at 1000× and 100 fibers were measured for each sample.

Fork velocity was determined by the total length of fibers (IdU plus CldU) divided by 40 min. The above listed protocol was followed for all experiments except for changes in the labeling scheme as listed below: For experiments where DMSO or HDIs were added after labeling with IdU followed by CldU, cells were labeled with IdU for 20 mins followed by 20 mins of CldU, washed, and then either immediately treated with DMSO or HDIs for 25 mins or incubated in fresh medium for 4 hrs and then treated with DMSO or HDIs for 25 mins. Fork Velocity was determined by the total length of fibers (IdU plus CldU) divided by 40 min pulse or by the length of either the IdU label or CldU label divided by 20 min pulse.

## Results

### Selectivity of Novel Histone Deacetylase Inhibitors

**Figure 1:**
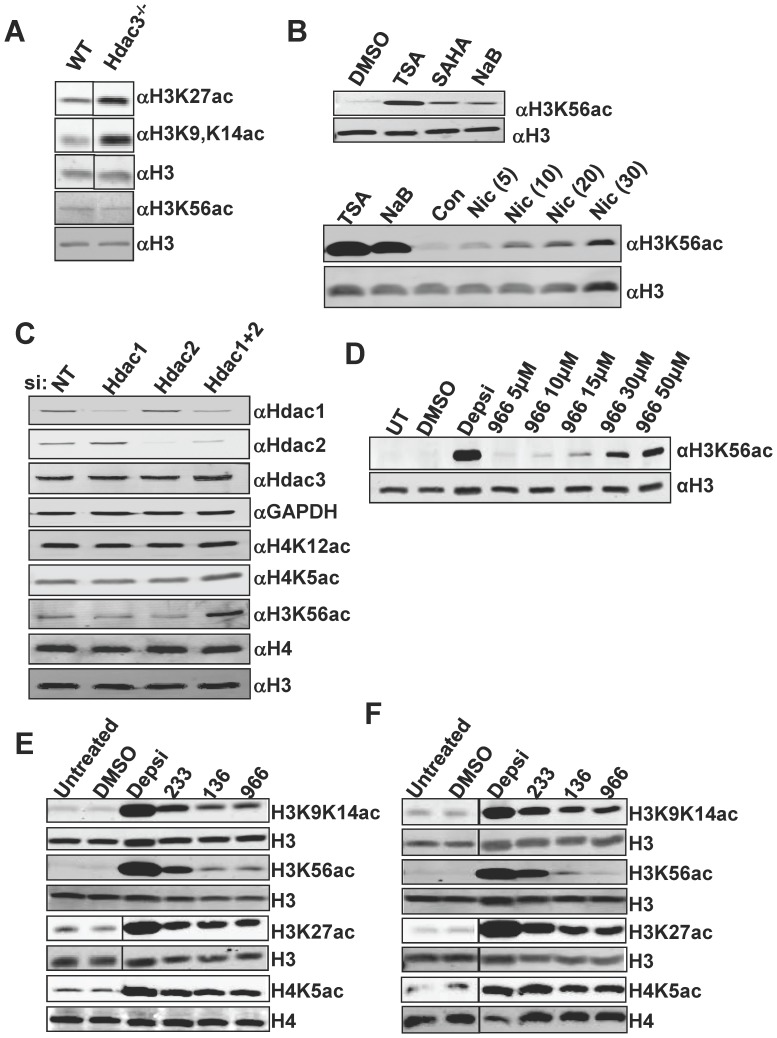
HDIs show selective inhibition of HDACs in CTCL cell lines. (A)Western blot analysis of whole cell lysates from Wild-type (WT) and *Hdac3*-null livers. Histones H3 and H4 served as loading controls. (B) Upper Panel: Western blot analysis of NIH 3T3 cells following treatment with various HDIs (indicated above each lane). Anti-histone H3 was used as a loading control. Lower panel: Western blot analysis of NIH 3T3 cells treated with either Trichostatin A (TSA) (1 µM), sodium butyrate (NaB) (5 mM), or increasing concentrations of nicotinamide (mM). (C) Western blot analysis of whole cell lysates prepared from cells that were transfected with either non-targeting siRNAs (NT) or siRNAs directed to the indicated Hdacs. (D) Western blot analysis of H3K56ac using whole cell lysates prepared from cells treated with the indicated amounts of RGFP966 for 24 hr. (E & F) Western blot analysis of (E) HH or (F) Hut78 cell lines treated with DMSO, 10 nM Depsipeptide (Depsi), 10 µM 233, 10 µM 136, or 10 µM 966. Cells were treated for 24 hr and then harvested for protein isolation. Samples were run on the same gel and probed on the same membrane. Intervening lanes (represented by a black bar) were removed for side-by-side comparison of DMSO and Depsipeptide. Histones H3 and H4 were used as loading controls.

The development of selective class I HDAC inhibitors has been challenging due to the conservation of the deacetylase domains of HDACs1-3, yet recently some selectivity has been achieved [32]–[34], [38]. To further assess the action of these inhibitors, we sought a histone mark that separates the functions of HDAC1/2 from HDAC3. Deletion of *Hdac3* caused increases in the acetylation of H4K5, H4K8, H4K12, H4K16, H3K9K14, and H3K27 [16], which are also targeted by Hdac1/2 [39]. However, we noted that *Hdac3* deletion did not cause the accumulation of the modification recognized by the rabbit monoclonal antibody to H3K56ac (Figure 1A). While this antibody can also cross react with H3K9ac [40], anti-H3K9ac did increase in *Hdac3^−/−^* cells, suggesting that under the conditions used here we did not detect H3K9ac with this antibody (Figure 1A; note that all samples were run on the same gel, but we removed intervening lanes for side by side comparison of WT and *Hdac3^−/−^* samples). In contrast, inhibitors of class I HDACs (SAHA, Trichostatin A and sodium butyrate (NaB)), caused a more dramatic accumulation of H3K56ac than nicotinamide, which impairs the Sirtuins (Figure 1B). Therefore, we used siRNAs directed to *Hdac1* and *Hdac2* and found that co-suppression of the expression of both enzymes was necessary to cause H3K56ac to accumulate, suggesting that both of these enzymes can target this mark, but that Hdac3 fails to deacetylate this residue (Figure 1C).

Given that H3K56ac separates the action of HDAC1/2 from HDAC3, we tested selective Hdac1/2 (RGFP233) and Hdac3 selective inhibitors (RGFP136 and RGFP966) for specificity. RGFP233 (233) showed 100- and 50-fold selectivity respectively towards HDAC1 and HDAC2 over HDAC3, and RGFP136 (136) and RGFP966 (966) were 10- and >100-fold respectively more selective for HDAC3 in *in vitro* deacetylase assays [32] [Vincent Jacques, Repligen unpublished data]. A titration of RGFP966 showed that at 5–10 µM there was only a modest affect on H3K56ac, which was approximately 15-fold less than found with Depsipeptide (Fig. 1D). Treatment of two CTCL cell lines, HH and Hut78, with the HDAC3-selective inhibitors 966 and 136, for 24 hours prior to western blot analysis resulted in increased acetylation at H3K9/K14, H3K27, and H4K5, but not H3K56ac (even at 10µM, Figure 1E and F). In contrast, Depsipeptide, an inhibitor of the class I HDACs (HDACs 1, 2, 3, and 8) [10], [13], caused the robust accumulation of all of the histone acetylation marks tested, whereas the HDAC1/2-selective inhibitor, 233, caused a less robust accumulation of these same marks. Using the Odyssey imaging system, we measured the fluorescence (integrated intensity units) of each band and found that 966 and 136 were at least 8–10-fold selective for HDAC3 inhibition by these criteria, even when used at relatively high levels (Figure 1E and F), confirming the *in vitro* data that 136 and 966 are selective for HDAC3 inhibition [Vincent Jacques, Repligen unpublished data]. Importantly, 966 was determined to have no inhibition of other HDACs at concentrations up to 15 µM in *in vitro* assays [32], which is consistent with our finding of only modest increases in H3K56ac at 10 µM.

### HH and Hut78 CTCL Cell Lines Show Sensitivity to Novel, Selective HDIs and Additive Effects with CTCL Clinical Drugs

**Figure 2:**
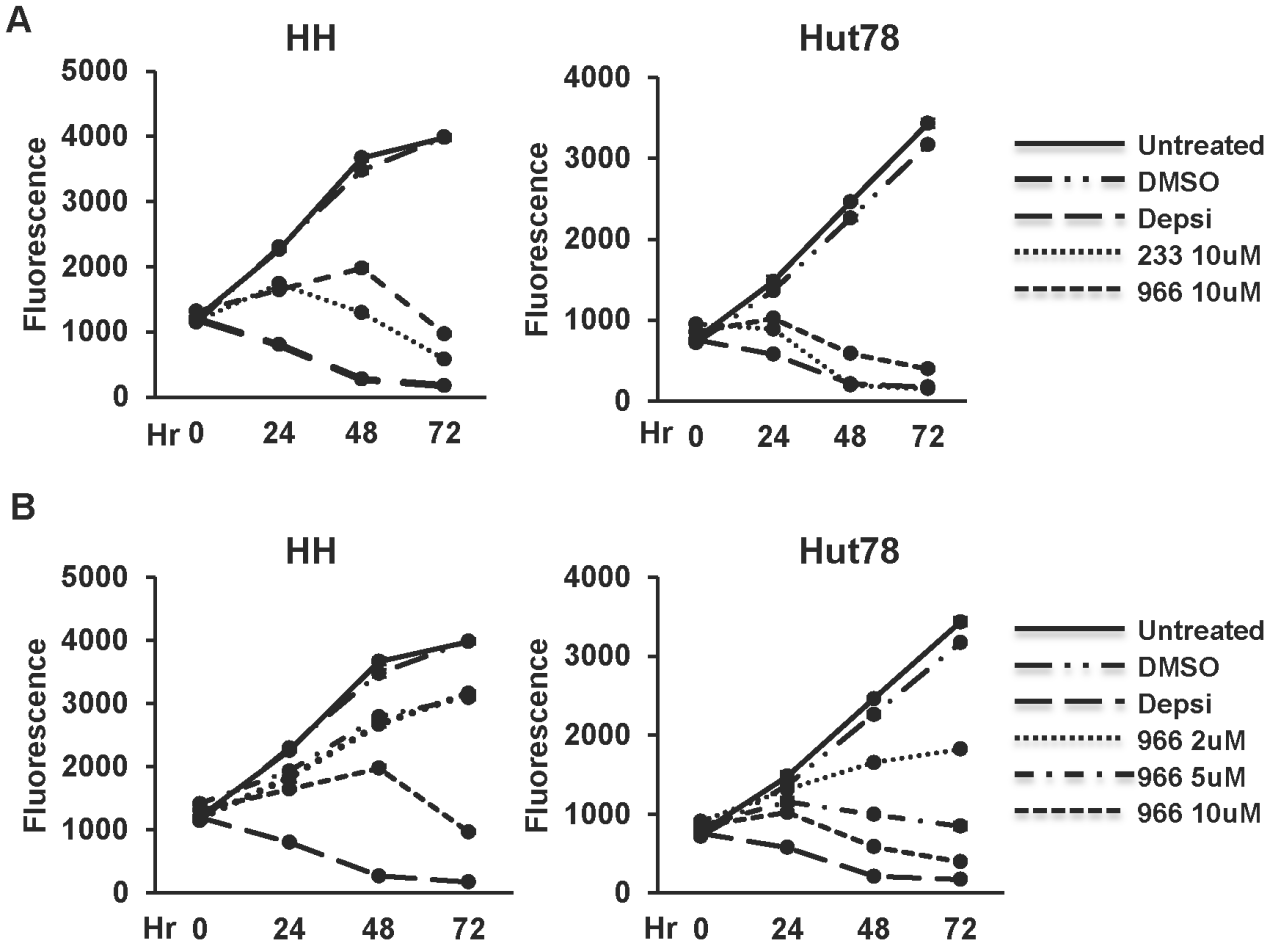
CTCL cell lines are sensitive to pan and selective HDIs. (A) Growth curves of HDI treated HH cells (left) or Hut78 cells (right). Cells were treated once with DMSO, 10 nM Depsipeptide (Depsi), 10 µM 233, or 10 µM 966 at hour 0. Untreated cells and DMSO treated cells were used as controls. Cell growth was assessed at 0, 24, 48, and 72 hours after treatment. (B) Dose curves of 966 treated HH cells (left) and Hut78 cells (right). The experiment was performed in the same manner as (A) except that the cells treated were treated once with 2 µM, 5 µM, or 10 µM of 966 at hour 0. For both (A) and (B), representative curves are shown from experiments performed in triplicate that are consistent with other biological replicates. Statistical analysis was performed using a two-tail paired T-test and comparing the HDI treated cells to DMSO treated cells resulting in the following p values: (A) HH cells (left), Depsi: p = 0.0008, 233: p = 0.004, and 966: p = 0.006. For the Hut78 cells (right), Depsi: p = 0.002, 233: p = 0.006, and 966: p = 0.006. (B) HH cells (left), Depsi: p = 0.0008, 966 2 µM: p = 0.02, 966 5 µM: p = 0.01, and 966 10 µM: p = 0.006. For the Hut78 cells (right), Depsi: p = 0.002, 966 2 µM: p = 0.03, 966 5 µM: p = 0.01, and 966 10 µM: p = 0.006.

To determine how treatment with selective HDIs affects CTCL cell lines, we first performed cell proliferation assays using alamar blue to measure cell growth and viability in the presence of different HDIs. HH and Hut78 cells were treated at hour 0 with either DMSO, Depsipeptide, 233, or 966 and then analyzed at hours 0, 24, 48, and 72 for changes in cell proliferation as measured by changes in alamar blue-dependent fluorescence. Both cell lines were sensitive to treatment with 10 µM 233 or 966, as demonstrated by decreases in cell growth over time (Figure 2A). However, Hut78 cells exhibited a greater sensitivity to these HDIs than HH cells. Neither cell line was affected by the DMSO control, and Depsipeptide, which targets all class 1 HDACs was very efficient at cell killing. Therefore, we tested the combined effects of 233+966 and found additive effects, consistent with the selective targeting of HDAC1/2 and HDAC3 by these compounds (Fig. S1).

Dose curves were performed on each cell line to determine the optimal dose for dual treatment with drugs that are used or have been used to treat CTCL (Figure 2B). Cells were treated with varying concentrations of 233, 136, or 966 at hour 0 and again analyzed using alamar blue cell viability assays. CTCL cells showed dramatic sensitivity to 233 at each concentration, with Hut78 cells again exhibiting heightened sensitivity when compared to HH cells (Figure S2A). Treatment of cells with 136 had only modest effects on cell growth when compared to treatment with 966 (Figure S2B and Figure 2B) in both cell lines. Thus, we discontinued the analysis of 136 in subsequent experiments and focused on the inhibition of Hdac3 using 966.

**Figure 3:**
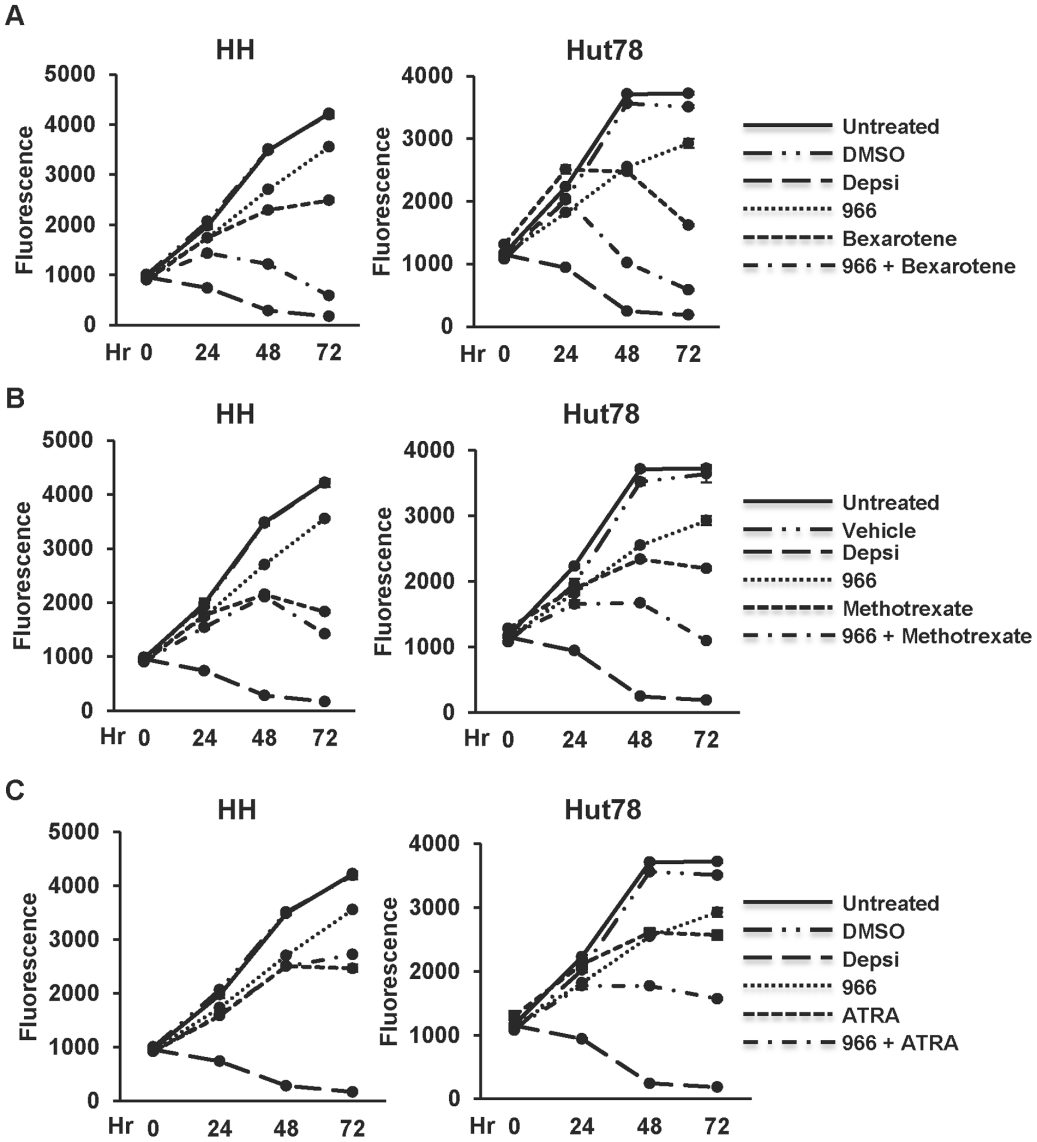
Dual treatment with RGFP966 and CTCL drugs has an additive effect on cell growth. Growth curves of dual treatment on HH cells or Hut78 cells. Cells were treated once at hour 0 with DMSO, 10 nM Depsipeptide (Depsi), 2 µM 966, or a combination of 2 µM 966 and either Bexarotene, Methotrexate, or ATRA. Untreated cells and DMSO treated cells were used as controls. Cell growth was assessed at 0, 24, 48, and 72 hours after treatment. (A) HH cells (left) or Hut78 cells (right) were treated with 20 µM or 75 µM Bexarotene alone or in combination with 966. (B) Cells were treated with 0.1 µM Methotrexate alone or in combination with 966. DMSO and 1 M Na_2_CO_3_ served as vehicle controls. (C) Cells were treated with 2 µM ATRA alone or in combination with 966. ATRA was administered at hour 0 and re-dosed at 48 hours after treatment. For (A–C), representative curves are shown from experiments performed in triplicate that are consistent with other biological replicates. Statistical analysis was performed using a two-tail paired T-test and comparing the HDI, CTCL drug, or dual treated cells to DMSO treated cells resulting in the following p values: (A) HH cells (left), Depsi: p = 0.0008, 966: p = 0.003, Bexarotene: p = 0.003, and 966 plus Bexarotene: p = 0.002. For the Hut78 cells (right), Depsi: p = 0.001, 966: p = 0.08, Bexarotene: p = 0.01, and 966 plus Bexarotene: p = 0.009. (B) HH cells (left), Depsi: p = 0.0008, 966: p = 0.003, Methotrexate: p = 0.003, and 966 plus Methotrexate: p = 0.003. For the Hut78 cells (right) Depsi: p = 0.001, 966: p = 0.01, Methotrexate: p = 0.01, and 966 plus Methotrexate: p = 0.004. (C) HH cells (left), Depsi: p = 0.0008, 966: p = 0.003, ATRA: p = 0.002, and 966 plus ATRA: p = 0.0007. For the Hut78 cells (right) Depsi: p = 0.001, 966: p = 0.01, ATRA: p = 0.02, and 966 plus ATRA: p = 0.004.

A number of therapies are currently used for the treatment of CTCL and given that single agent therapy is rarely beneficial, we tested Bexarotene (highly selective retinoid x receptor agonist), Methotrexate (inhibitor of dihydrofolate reductase), or ATRA (All Trans Retinoic Acid, a retinoic acid receptor agonist) [1], [41]–[43] for cooperative cell killing with 966. A dose of 2 µM for 966 was selected for dual treatment experiments so that we could assess additive or synergistic effects when 966 was combined with these drugs. Dose curves for Bexarotene, Methotrexate, and ATRA were performed and concentrations near the IC_50_ were chosen (Figure S3). Both HH and Hut78 cells exhibited increased sensitivity to dual treatment of 966 plus Bexarotene (Figure 3A), while only Hut78 cells showed increased sensitivity to 966 plus Methotrexate or ATRA (Figure 3B and C).

### CTCL Cell Lines Undergo Apoptosis, have Increased DNA Damage, and Exhibit Cell Cycle Defects

**Figure 4:**
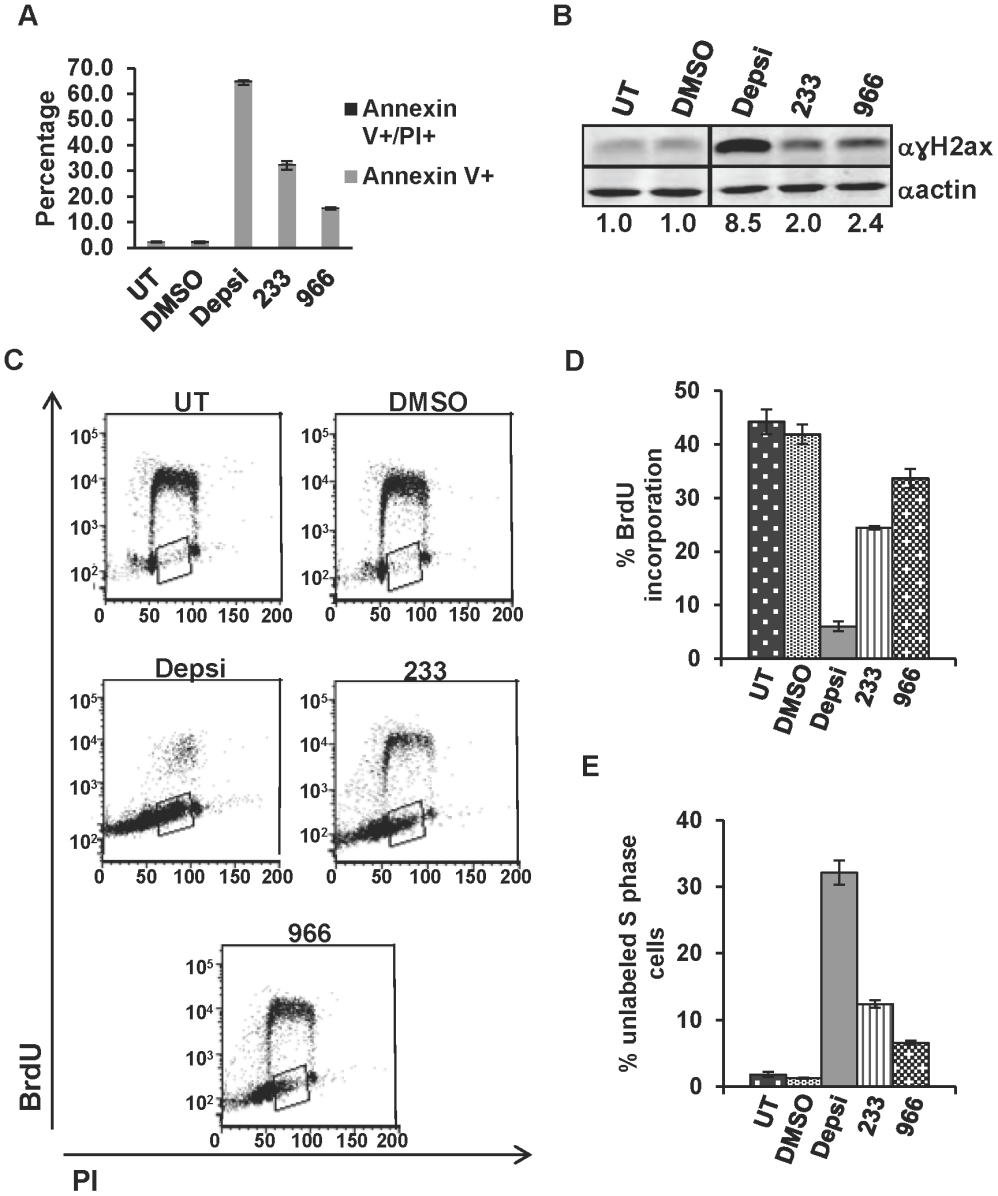
An HDAC3 selective inhibitor triggers apoptosis associated with increased DNA damage and cell cycle defects. (A) Hut78 cells were treated with DMSO, 10 nM Depsipeptide (Depsi), 10 µM 233, or 10 µM 966 for 24 hr and apoptosis assessed by Annexin V staining and flow cytometry. Cells were also labeled with propidium iodide to assess DNA content. Untreated (UT) and DMSO treated cells were used as controls. Shown is a representative graph from an experiment performed in duplicate that is consistent with other biological replicates. (B) Western blot analysis of γH2aX levels in Hut78 cells treated with DMSO, 10 nM Depsi, 10 µM 233, or 10 µM 966 for 8 hrs. Untreated and DMSO treated cells were used as controls. Samples were run on the same gel and probed on the same membrane. Intervening lanes (represented by a black bar) were removed for side by side comparison of DMSO and Depsipeptide. (C) Cell cycle status was analyzed using BrdU incorporation and propidium iodide to assess DNA content by flow cytometry. Hut78 cells were treated with DMSO, 10 nM Depsipeptide (Depsi), 10 µM 233, or 10 µM 966 for 24 hr and pulsed for an hour and a half with BrdU prior to cell harvest and analysis. Shown are representative flow cytometry plots from an experiment performed in duplicate that is consistent with other biological replicates. (D) Graphical representation of BrdU incorporation from the experiment described in (C). (E) Graphical representation of the percent of S phase cells that did not incorporate BrdU (shown by box in panel (C)). Statistical analysis for both the Annexin V and BrdU experiments was performed using a two-tail T-test and comparing the HDI treated cells to the DMSO treated cells resulting in the following p-values: (A) Depsi: p = 0.0002, 233: p = 0.003, and 966: p = 0.0003. (D) Depsi: p = 0.003, 233: p = 0.01, and 966: p = 0.08. (E) Depsi: p = 0.003, 233: p = 0.003, and 966: p = 0.004.

We next determined whether the decreased cell growth seen when HH and Hut78 cells were treated with selective HDIs (Figures 2 and 3) was due to increased apoptosis. Flow cytometry analysis using Annexin V versus propidium iodide (PI) was performed on HH and Hut78 cells that had been treated for 24 hours with DMSO, 10 nM Depsipeptide, 10 µM 233, or 10 µM 966. HH and Hut78 cells displayed significant increases in Annexin V levels following treatment with HDIs, with Hut78 cells exhibiting the highest Annexin V levels (Figure 4A and Figure S4). Therefore, these cells undergo apoptosis when treated with HDIs. In both cell lines, Depsipeptide treatment resulted in the greatest cell killing, followed by 233 and 966. This trend may reflect the fact that Depsipeptide inhibits all three class I HDACs, 233 inhibits two HDACs, and 966 selectively inhibits a single HDAC.

Deletion of *Hdac3* caused increased DNA damage and cell cycle delays in an S phase dependent manner in fibroblasts [17]. To determine if the apoptosis occurring in Hut78 and HH cells when cells were treated with HDIs was associated with increased DNA damage, we treated cells for 8 hours with DMSO, Depsipeptide, 233 or 966 and performed western blot analysis using anti-γH2aX, which is localized to sites of DNA double-strand breaks [44]. Both cell lines showed approximately a 2.4-fold increase in the amount of γH2aX in samples treated with 966, indicative of an increase in DNA damage when HDAC3 was inhibited in CTCL cells (Figure 4B and Figure S3B). Treatment with Depsipeptide or 233 also caused increased γH2aX levels in both cell lines, with Depsipeptide being the most robust. When HH and Hut78 cells were treated with DMSO, Depsipeptide, 233, or 966 for 24 hours and pulsed with BrdU for 90 min before harvest, Hut78 cells treated with HDIs exhibited decreased BrdU incorporation, and also an increase in cells that were present in S phase but were not incorporating BrdU (Figure 4C–E and Figure S3C–E). These S phase cells that did not incorporate BrdU represent cells that have not completed DNA replication and are arrested in the S phase, suggesting that HDI treatment caused replication stress in CTCL cell lines.

### Inhibition of Hdac3 leads to DNA Replication Defects

**Figure 5:**
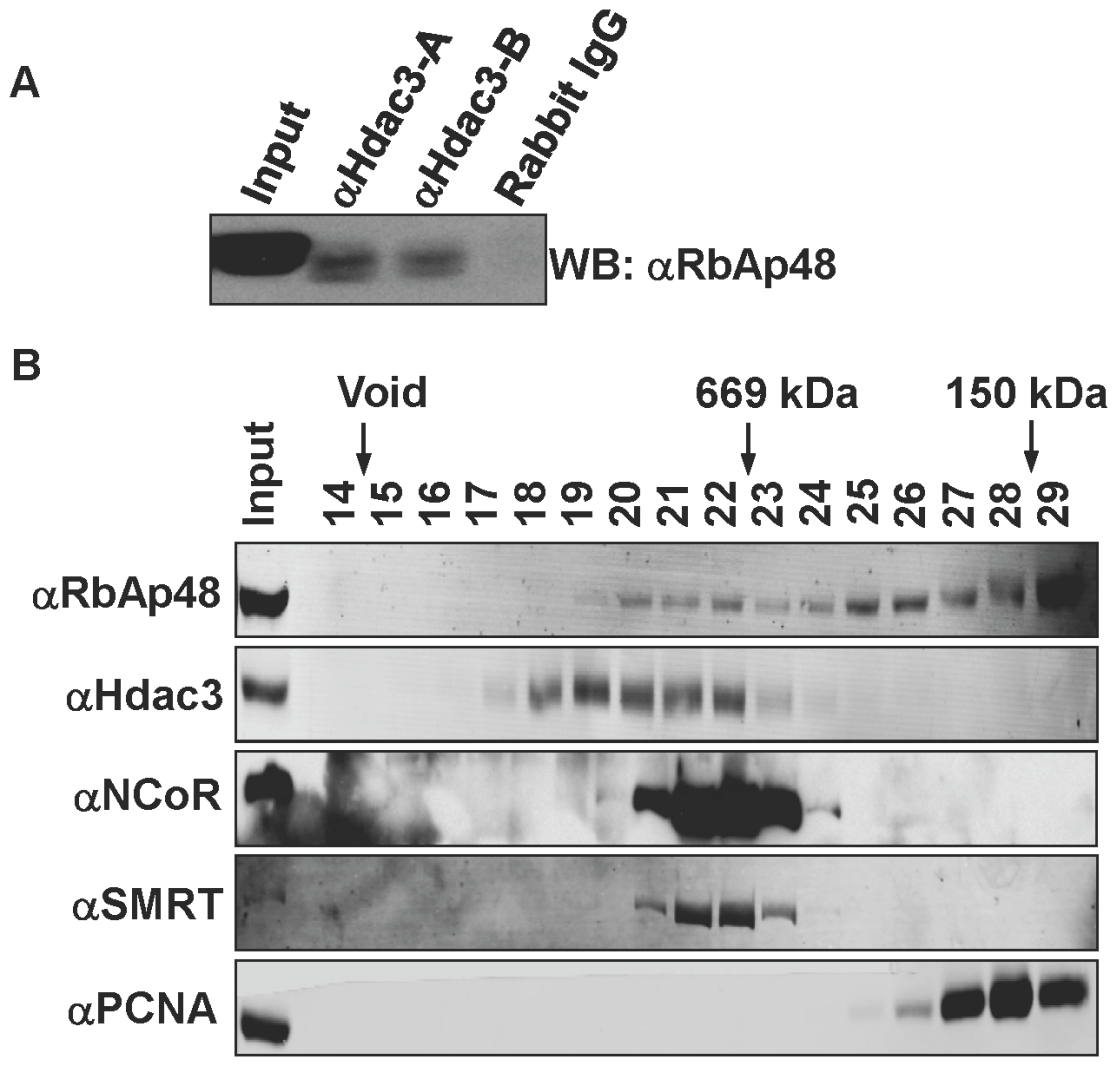
Hdac3 co-purifies with the histone chaperone, RbAp48, in mammalian cells. (A) Immunoprecipitation analysis of endogenous HDAC3 and RbAp48 from HeLa cells. Two different HDAC3 antibodies were used and labeled (A) or (B) and rabbit IgG was included as a negative control. (B) Gel Filtration analysis of HDAC3 containing protein complexes. Nuclear lysates were separated using a Superose 6 gel filtration column and the elution profile of the indicated proteins determined by western blot analysis. The elution of size markers is shown at the top of the blots.

HDACs 1 and 2 regulate deacetylation of histones deposited on newly synthesized DNA during S phase and are enriched at replication forks [16], [39], [45] through association with histone chaperones like RbAp48 and CAF1 [25], [46]–[48]. Like HDAC1 and 2, HDAC3 also targets histone deposition marks ([16] and Figure 1), and yeast two-hybrid studies show that HDAC3 can also bind to RbAp48 [49]. Therefore, we tested whether HDAC3 could associate with RbAp48 in mammalian cells. Immunoprecipitation analysis of endogenous HDAC3 and RbAp48 from HeLa cells detected an association, suggesting that HDAC3 could be bound to histone chaperones on chromatin (Figure 5A). To extend this analysis, we used gel filtration to determine the sizes of native HDAC3-containing complexes (Figure 5B). HDAC3 co-eluted with a portion of the RbAp48, but not PCNA, which marks DNA replication complexes (Figure 5B).

**Figure 6:**
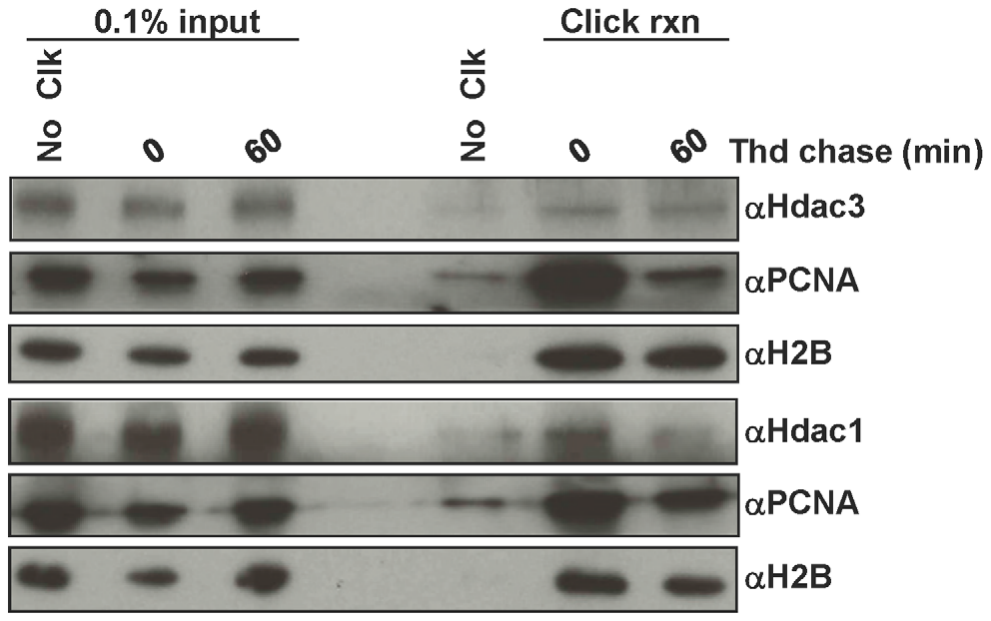
iPOND analysis reveals HDAC3 association with replication forks in Hut78 CTCL cells. Hut78 cells were pulsed for 15 minutes with EdU followed by either no thymidine chase or a 60 minute thymidine chase. The protein-DNA complexes were then cross-linked, nascent DNA was conjugated to biotin using click chemistry, and then protein-DNA complexes were purified using Streptavidin beads. The eluted proteins were then analyzed using western blot analysis. A no click reaction sample (No Clk) that did not include biotin azide was used as a negative control. 0.1% input samples were included for positive controls of each protein analyzed. PCNA served as a positive control for a replication fork bound protein and H2B served as a loading control and positive control for a chromatin bound protein.

The gel filtration analysis suggested that HDAC3 might be associated with histone deposition machinery, yet not directly bound to the DNA replication machinery. Therefore, isolation of proteins on nascent DNA (iPOND) was used to further probe HDAC3 localization to DNA replication forks. A similar analysis in HEK293T cells suggested that, not only were HDAC1 and HDAC2 present at DNA replication forks, but HDAC3 was also detected [45]. To test whether HDAC3 was also present at replication forks in CTCL cells, Hut78 cells were pulsed for 15 minutes with EdU (5-Ethynyl-2′-deoxyuridine) only or pulsed with EdU for 15 minutes followed by a 60 minute thymidine chase. After the labeling, cells were cross-linked, and the nascent DNA with EdU incorporated was conjugated to biotin using click chemistry. The newly synthesized DNA and the DNA-protein complexes were then purified using streptavidin beads. Proteins that move with the replication fork such as HDAC1 and PCNA [36], [45] were enriched immediately after EdU labeling (lanes labeled “0”, Figure 6) and then decreased with the thymidine chase. By contrast, western blot analysis showed that HDAC3 was bound to chromatin at and around replication forks, but like H2B, its levels did not significantly drop after the 60 minute chase, suggesting that it did not travel with replication forks (Figure 6).

**Figure 7:**
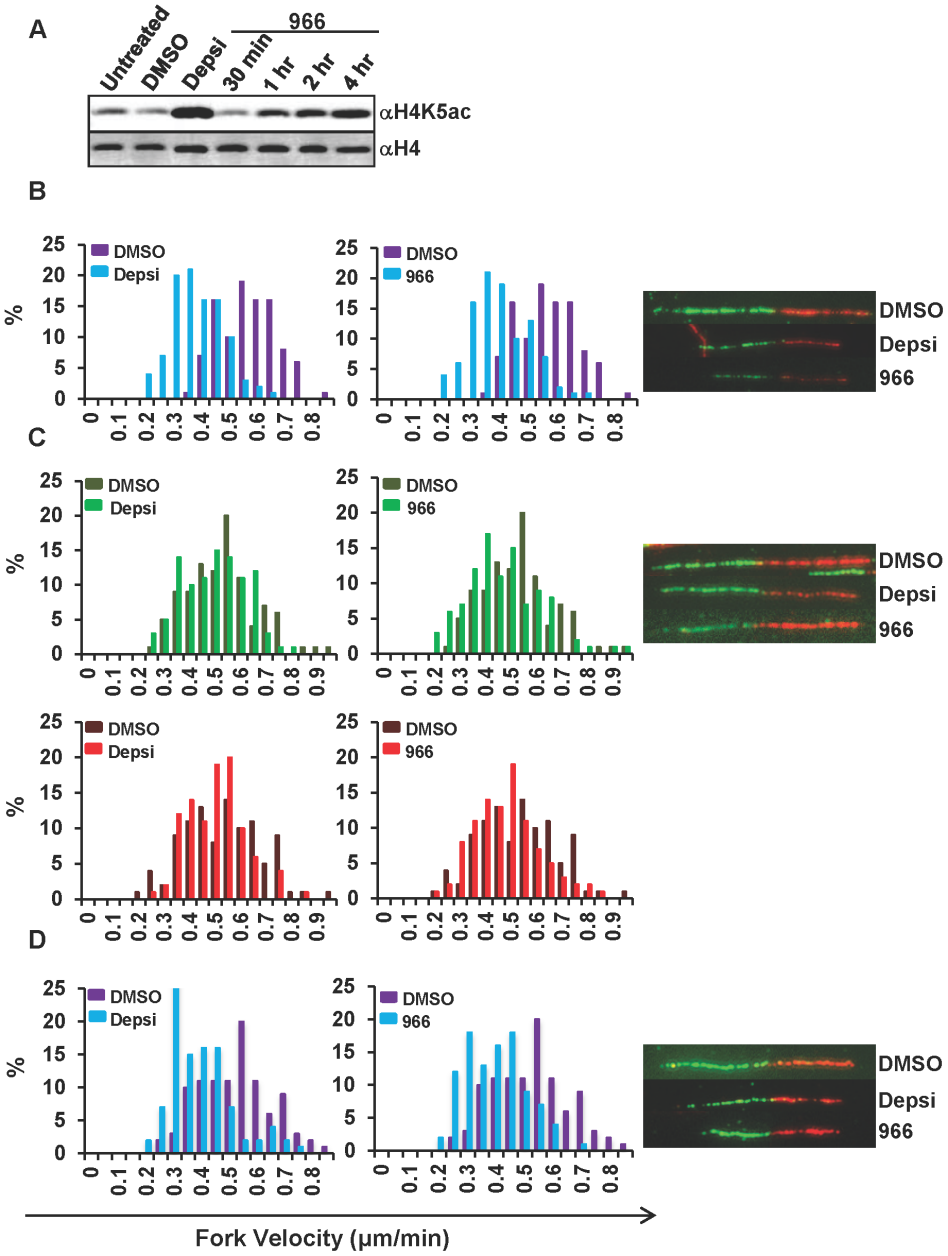
HDAC3 selective inhibitors rapidly cause defects in DNA replication. (A) Western blot analysis of Hut78 cells treated with DMSO or 10 nM Depsipeptide (Depsi) for 4 hrs, or 10 µM 966 for 30 min, 1 hr, 2 hr, and 4 hr. (B, C, and D) DNA fiber labeling analysis was used to assess DNA replication fork progression in Hut78 cells treated with DMSO, 10 nM Depsipeptide (left) or 10 µM 966 (right) for 4 hr (B) or 5 mins (D) prior to labeling with 20 mins of IdU (green) followed by 20 min of CldU (red). Graph of fork velocity (length of fibers divided by 40 min) is shown. (C) Hut78 cells were treated with DMSO, Depsi or 966 immediately after labeling cells with IdU followed by CldU. Graph of fork velocity for either the IdU label or CldU label is shown. Representative fibers are shown. 100 fibers were measured for each sample. Statistical analysis was performed using Mann-Whitney test and standard deviations were calculated. HDI treated cells were compared to DMSO treated cells resulting in the following p-values: (B) Depsi: p<0.0001; 966: p<0.0001. The average velocities for Depsi and 966 were greater than 3 standard deviations of the DMSO average velocity. (C) Depsi IdU (green): p = 0.1, Depsi CldU (red): p = 0.1; 966 IdU (green): p = 0.0011; 966 CldU (red): p = 0.01. The average velocities for IdU and CldU in Depsi treated cells were within 1 and 2 standard deviations respectively of the DMSO average velocity. The average velocities for IdU and CldU in 966 treated cells were within 2 standard deviations of the DMSO average velocity. (D) Depsi: p<0.0001; 966: p<0.0001. The average velocities for Depsi and 966 were greater than 3 standard deviations of the DMSO average velocity.

Although HDAC3 did not appear to move with replication forks using iPOND, loss of HDAC3 activity using siRNA or gene deletion showed a requirement for this deacetylase for optimal DNA replication fork velocity [[50],Summers,unpublished data]. A major advantage of small molecules is that they allow the analysis of HDAC function in short timeframes that cannot be replicated by genetic methods. We started by assessing the minimal time required to achieve HDAC3 inhibition using 966. Hut78 cells were treated with DMSO, Depsipeptide, or 966 for 30 min, 1 hr, 2 hr, and 4 hr and western blot analysis for H4K5ac was used as a measure of HDAC3 inhibition (Figure 7A). In purified enzyme assays, 966 is a slow on/slow off inhibitor when used at nanomolar concentrations, where full potency was observed within approximately 2 hr. Treatment with 10 µM 966 for 30 min did not significantly increase H4K5 acetylation levels, but by 1 hr a noticeable increase in H4K5 acetylation was apparent, and by 4 hr a dramatic accumulation of H4K5 acetylation was observed (Figure 7A) suggesting full inhibition within 4 hr. This suggests that HDAC3-regulated histone acetylation is very dynamic with changes in histone acetylation detectable by western blot occurring within hours of treatment, but within 30 min of Hdac3 inhibition by 966 there were not global effects on histone acetylation.

Next, DNA fiber labeling analysis was used to visualize individual DNA fibers by sequential labeling of cells with IdU and CldU followed by immunofluorescence to detect the incorporation of these analogs [37] in strands of DNA to measure replication fork velocity. Treatment with Depsi or 966 for 4 hrs prior to labeling with IdU followed by CldU resulted in a shortening of the average length of fiber tracks (examples of fibers are shown on the right), which corresponds to slower replication fork progression than the DMSO control (Figure 7B). To ensure that changes in chromatin structure did not affect fiber track length after replication fork progression, which would interfere with accurate measurement of DNA fibers, Hut78 cells were labeled with IdU followed by CldU, washed and then were either immediately treated with DMSO or HDIs for 25 min or incubated in fresh medium for 4 hr and then treated with DMSO or HDIs for 25 min. Neither of these experiments showed significant changes in fiber track length or fork velocity (Figure 7C & S5), confirming that the effects on replication seen with inhibition of HDAC3 are not due to shortening of fiber track lengths due to global changes in chromatin structure.

Finally, to determine if this replication defect was due to a localized effect, we treated Hut78 cells for 5 min with either Depsi or 966 before labeling with IdU followed by CldU. Remarkably, even treatment within this short timeframe caused a shortening of DNA fiber track lengths and slower fork velocity (Figure 7D). These data suggest that treatment with a HDAC3 selective inhibitor has localized effects on replication at or nearby the replication fork since global changes in H4K5ac were not seen within 30 min of treatment with 966 (Figure 7A).

## Discussion

Cutaneous T cell lymphoma (CTCL) diagnosed during early stage disease generally has an indolent course and good outcome [1]–[5]. However, late stage, refractory, or aggressive CTCL (such as Sézary Syndrome) has a shortened survival expectancy [1]–[5]. Two histone deacetylase inhibitors, SAHA and Depsipeptide, have been FDA approved for the treatment of late stage or refractory CTCL [1], [3], [10]–[12]. However, since these HDIs target multiple HDACs, it is unknown which of these HDACs must truly be inhibited to achieve the anti-tumor effects observed upon HDI treatment. Furthermore, it is likely that the unnecessary inhibition of other HDACs contributes to the side effects seen with HDI treatment (such as nausea, fatigue, and GI, cardiac and hematologic toxicities). By using selective HDIs, the efficacy of individual HDAC targeting can be assessed and side effects may be lessened, resulting in improved quality of life for patients undergoing treatment. Here, we show that the inhibition of HDAC1/2 or HDAC3 through the use of novel, selective inhibitors, caused decreased cell growth of the CTCL cell lines, HH and Hut78 by triggering apoptosis (Figure 2). While it appears that inhibition of all three of these HDACs was more efficacious (e.g., Depsipeptide worked very well), more potent selective inhibitors may yield better results, or the lower toxicity may allow more intensive or longer-term treatments. Ultimately, having HDAC1/2 versus HDAC3 selective inhibitors will provide flexibility in defining the best schedules and combinations of these compounds to maximize the therapeutic benefit in the treatment of CTCL.

Mechanistically, the apoptosis observed was associated with the accumulation of DNA damage in HDI treated cells (Figure 4 and S3). BrdU-labeling studies showed decreased BrdU incorporation with pan HDAC inhibitors, inhibitors of HDAC1/2 and the HDAC3 selective inhibitors (Figure 4 C–E). These studies also revealed a significant increase in cells that did not incorporate BrdU, but showed increased DNA content, consistent with an S-phase arrest following HDI treatment, suggesting that the DNA damage was due to defects in DNA replication. This prompted an analysis of DNA replication fork velocity using DNA fiber labeling assays, which showed that Depsipeptide treatment and treatment with the Hdac3 selective inhibitor resulted in inefficient or slowed DNA replication (Figure 7). By examining DNA replication shortly after adding the HDIs, we were able to show that this is a very early event, occurring within the first hour of HDI treatment. These data suggest that HDI therapy first affects DNA replication (Figure 7), which would provide a therapeutic window by targeting the cycling cancer cells, and leaving normal, non-cycling cells intact.

The rapid effects of 966 on DNA replication suggest an important role for HDAC3 in DNA replication. In addition, by inhibiting HDAC3 at various times before DNA fiber labeling, we were able to narrow the possible mechanisms by which this might occur to localized effects at or around the DNA replication fork, as it took greater than 30 min before global changes in histone acetylation were observed (Figure 7). However, these studies cannot discriminate whether this is due to a local chromatin effect or whether HDAC3 directly targets the DNA replication machinery. For instance, chromatin in and around the DNA replication fork must be in an open configuration, which is more accessible to HDAC3 than nucleosomes in mature chromatin. Because the histones in newly placed nucleosomes are acetylated prior to deposition, inhibition of HDAC3 could cause the accumulation of acetylation of these histones within minutes of HDI treatment, whereas global accumulation of H4K5ac takes an hour or more (Figure 7A). Alternatively, components of the DNA replication machinery may be regulated by acetylation and deacetylation and HDAC3 could play a regulatory role. One argument against this is that HDAC3 did not co-elute with PCNA in size exclusion chromatography (Figure 5) or move with the DNA replication fork in iPOND purifications (Figure 6). Thus, at this point in time, the evidence best supports a localized effect on chromatin at the replication fork.

Although endogenous HDAC3 can associate with histone chaperones such as RbAp48 (Figure 5), its role in deacetylation of newly formed nucleosomes is largely based on genetic, siRNA and chemical inhibition studies ([16], [17] and Figure 7). These studies indicate that HDAC3 targets the same histone deposition marks that HDAC1/2 deacetylate and that HDAC3 is required at replication forks (Figure 6, 7). Historically, HDAC1/2 were biochemically linked to histone deposition [16], [39]. These enzymes form nearly stoichiometric complexes with the histone deposition machinery and are thought to be the major enzymes responsible for the deacetylation of new nucleosomes. Moreover, siRNA or genetic impairment of HDAC1 is compensated by higher expression of HDAC2 (e.g., Figure 1C), whereas deletion of *Hdac3* is not compensated for by higher expression of other class 1 Hdacs. Thus, we conclude that HDAC3 plays a distinct role from HDAC1 and HDAC2 during chromatin maturation (Figure 6) and that targeting HDAC3 with small molecule inhibitors will provide additional therapeutic impact in the treatment of CTCL and other cancers.

Currently, SAHA and Depsipeptide are approved as single agents to treat refractory CTCL [1], [3], [10]–[12]. However, combinatorial treatment is almost always more beneficial than single agent therapy, so we tested HDAC3 inhibitors with other drugs currently used for CTCL. The combination of 966 and either bexarotene, methotrexate, or ATRA led to further reductions in cell growth than either agent alone in Hut78 cells (Figure 3), but these effects were additive, not synergistic. Nevertheless, these combinations did not negate the responses of these drugs, suggesting that these compounds could be used together in the clinic. Our studies show that individual HDACs can be targeted and that these inhibitors may be useful in the treatment of CTCL by rapidly targeting DNA replication. While the first effects of these compounds may be at replication forks (which provides a therapeutic window), within only 4 hr these drugs also affected global histone acetylation, which indicates that HDAC3 plays a dynamic role in the regulation of histone acetylation and chromatin structure. Thus, these compounds may target multiple fundamental events in the cell cycle to trigger apoptosis in cycling tumor cells that would be beneficial in combination with current therapies for CTCL.

## Supporting Information

Figure S1 **CTCL cell lines exhibit additive sensitivity to the combination of 233 and 966.** Viability curves of Hut78 cells treated with the indicated amounts of RGFP966 and 233. Cells were treated once with DMSO, 10 nM Depsipeptide (Depsi), or different concentrations of either 233 or 966 at hour 0. Untreated cells and DMSO treated cells were used as controls. Cell growth was assessed at 0, 24, 48, and 72 hours after treatment using alamar blue. A representative curve is shown from experiments performed in triplicate that are consistent with other biological replicates.
(TIFF)

Figure S2 **CTCL cell lines exhibit sensitivity to multiple doses of 233 and high dose 136.** Dose curves of HH cells (left) or Hut78 cells (right) treated with 10 µM 233 (A) or 966 (B). Cells were treated once with DMSO, 10 nM Depsipeptide (Depsi), or different concentrations of either 233 or 136 at hour 0. Untreated cells and DMSO treated cells were used as controls. Cell growth was assessed at 0, 24, 48, and 72 hours after treatment using alamar blue. For both (A) and (B), representative curves are shown from experiments performed in triplicate that are consistent with other biological replicates. Statistical analysis was performed using a two-tail paired T-test and comparing the HDI treated cells to DMSO treated cells resulting in the following p values: (A) HH cells (left), Depsi: p = 0.0008, 233 2 µM: p = 0.005, 233 5 µM: p = 0.005, and 233 10 µM: p = 0.004. For the Hut78 cells (right), Depsi: p = 0.002, 233 2 µM: p = 0.01, 233 5 µM: p = 0.005, and 233 10 µM: p = 0.006. (B) HH cells (left), Depsi: p = 0.001, 136 1 µM: p = 0.1, 136 5 µM: p = 0.1, and 136 10 µM: p = 0.006. For the Hut78 cells (right), Depsi: p = 0.001, 136 1 µM: p = 0.08, 136 5 µM: p = 0.02, and 136 10 µM: p = 0.005.
(TIFF)

Figure S3 **Dose curves for Bexarotene, Methotrexate, and ATRA reveal optimal concentrations for combination treatments.** Dose curves of Bexarotene (A), Methotrexate (B), and ATRA (C) treated HH cells or Hut78 cells. Cells were treated at hour 0 with DMSO, 10 nM Depsipeptide (Depsi), or varying concentrations of Bexarotene, Methotrexate, or ATRA. Cell growth was assessed at 0, 24, 48, and 72 hours after treatment. In all studies except for (A), the HH and Hut78 cells were treated with the same varying concentrations of CTCL drugs. HH cells were treated with 10, 20, or 50 µM of Bexarotene while Hut78 cells were treated with 50,75, or 100 µM of Bexarotene. In (B) DMSO and a solution containing Na_2_CO_3_ served as vehicle controls. (C) ATRA was administered at hour 0 and re-dosed at 48 hours after treatment. For (A–C), representative curves are shown from experiments performed in triplicate that are consistent with other biological replicates. Statistical analysis was performed using a two-tail paired T-test and comparing the HDI or CTCL drug treated cells to DMSO treated cells resulting in the following p values: (A) HH cells (left), Depsi: p = 0.0007; Bexarotene 10 µM: p = 0.001; Bexarotene 20 µM: p = 0.004; Bexarotene 50 µM: p = 0.001. Hut78 cells (right), Depsi: p = 0.002; Bexarotene 50 µM: p = 0.8; Bexarotene 75 µM: p = 0.1; and Bexarotene 100 µM: p = 0.04. (B) HH cells (left), Depsi: p = 0.001; Methotrexate 0.1 µM: p = 0.007; Methotrexate 1 µM: p = 0.01; Methotrexate 10 µM: p = 0.01; Methotrexate 100 µM: p = 0.006. Hut78 cells (right) Depsi: p = 0.001; Methotrexate 0.1 µM: p = 0.005; Methotrexate 1 µM: p = 0.006; Methotrexate 10 µM: p = 0.004; Methotrexate 100 µM: p = 0.004. (C) HH cells (left), Depsi: p = 0.001; ATRA 500 nM: p = 0.008; ATRA 1 µM: p = 0.002; ATRA 2 µM: p = 0.003. Hut78 cells (right) Depsi: p = 0.001; ATRA 500 nM: p = 0.02; ATRA 1 µM: p = 0.005; ATRA 2 µM: p = 0.006.
(TIFF)

Figure S4 **HDIs increased in apoptosis, DNA damage, and cell cycle defects in HH cells.** (A) HH cells were treated with DMSO, 10 nM Depsipeptide (Depsi), 10 µM 233, or 10 µM 966 for 24 hr and apoptosis levels were assessed by Annexin V/PI staining and flow cytometry. Untreated (UT) and DMSO treated cells were used as controls. Shown is a representative graph from an experiment performed in duplicate that is consistent with other biological replicates. (B) Western blot analysis of γH2aX levels in HH cells treated with DMSO, 10 nM Depsi, or 10 µM 966 for 8 hrs. Untreated and DMSO treated cells were used as controls. (C) Cell cycle status was analyzed using BrdU/PI and flow cytometry. HH cells were treated with DMSO, 10 nM Depsipeptide (Depsi), 10 µM 233, or 10 µM 966 for 24 hr and pulsed for an hour and a half with BrdU prior to cell harvest and analysis. Shown are representative flow cytometry plots from an experiment performed in duplicate that is consistent with other biological replicates. (D) Graphical representation of BrdU incorporation from the experiment described in (C). (E) Graphical representation of the percent of S phase cells that did not incorporate BrdU (shown by box in panel (C)). Statistical analysis for both the Annexin V and BrdU experiments was performed using a two-tail T-test and comparing the HDI treated cells to the DMSO treated cells resulting in the following p-values: (A) Depsi: p = 0.02, 233: p = 0.01, and 966: p = 0.06. (D) Depsi: p = 0.002, 233: p = 0.05, and 966: p = 0.3. (E) Depsi: p = 0.03, 233: p = 0.07, and 966: p = 0.8.
(TIFF)

Figure S5 **HDI treatment after labeling with IdU and CldU shows no changes in DNA fiber length.** (A) DNA fiber labeling analysis was used to assess DNA fiber length in Hut78 cells treated with either DMSO, 10 nM Depsipeptide (left) or 10 µM 966 (right) 4 hrs after labeling the cells with IdU for 20 mins (green) followed by 20 mins of CldU (red). (A) Graphical representation of fork velocity as determined by the total length of fibers (IdU plus CldU) divided by 40 min pulse is shown. Representative measured fibers are shown at the right for DMSO, Depsi, and 966. 100 fibers were measured for each sample. Statistical analysis was performed using Mann-Whitney test and standard deviations were calculated. HDI treated cells were compared to the DMSO treated cells resulting in the following p-values: Depsi: p = 0.5 and 966: p = 0.4. The average velocities for both Depsi and 966 were within 1 standard deviation of the average velocity for DMSO.
(TIFF)
