# Supplementary figures and images for: Inhibition of Histone Deacetylase 3 Causes Replication Stress in Cutaneous T Cell Lymphoma
Source: PLoS One. 2013 Jul 22;8(7):e68915. doi: 10.1371/journal.pone.0068915 (PMC3718806; doi:10.1371/journal.pone.0068915)

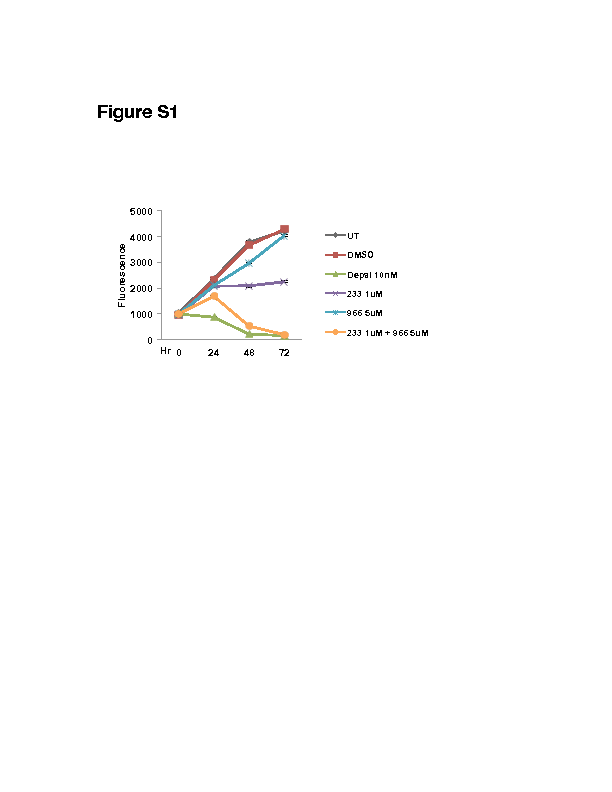

Supplement: Figure S1 — CTCL cell lines exhibit additive sensitivity to the combination of 233 and 966. Viability curves of Hut78 cells treated with the indicated amounts of RGFP966 and 233. Cells were treated once with DMSO, 10 nM Depsipeptide (Depsi), or different concentrations of either 233 or 966 at hour 0. Untreated cells and DMSO treated cells were used as controls. Cell growth was assessed at 0, 24, 48, and 72 hours after treatment using alamar blue. A representative curve is shown from experiments performed in triplicate that are consistent with other biological replicates. (TIFF) [file pone.0068915.s001.tiff]

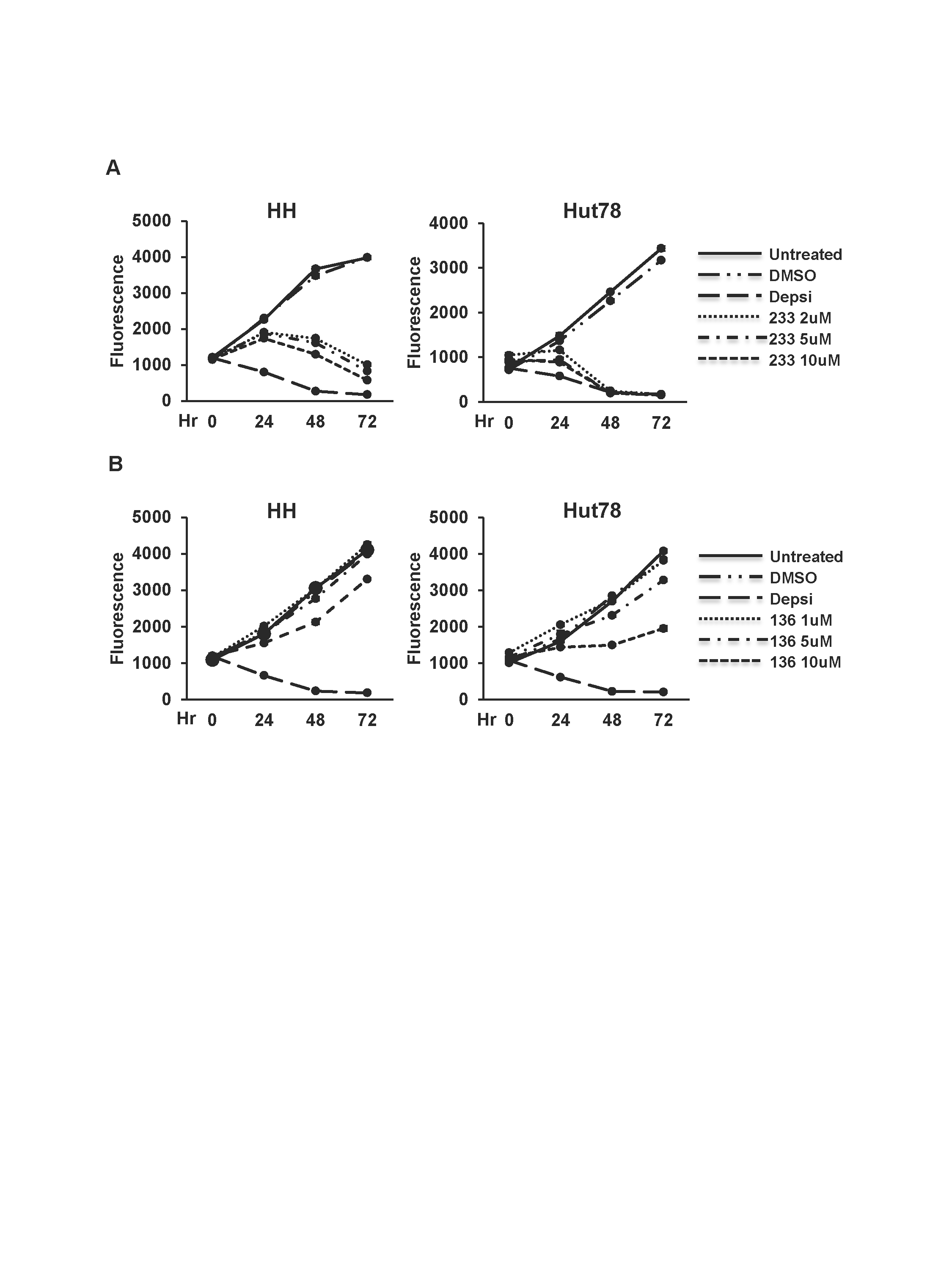

Supplement: Figure S2 — CTCL cell lines exhibit sensitivity to multiple doses of 233 and high dose 136. Dose curves of HH cells (left) or Hut78 cells (right) treated with 10 µM 233 (A) or 966 (B). Cells were treated once with DMSO, 10 nM Depsipeptide (Depsi), or different concentrations of either 233 or 136 at hour 0. Untreated cells and DMSO treated cells were used as controls. Cell growth was assessed at 0, 24, 48, and 72 hours after treatment using alamar blue. For both (A) and (B), representative curves are shown from experiments performed in triplicate that are consistent with other biological replicates. Statistical analysis was performed using a two-tail paired T-test and comparing the HDI treated cells to DMSO treated cells resulting in the following p values: (A) HH cells (left), Depsi: p = 0.0008, 233 2 µM: p = 0.005, 233 5 µM: p = 0.005, and 233 10 µM: p = 0.004. For the Hut78 cells (right), Depsi: p = 0.002, 233 2 µM: p = 0.01, 233 5 µM: p = 0.005, and 233 10 µM: p = 0.006. (B) HH cells (left), Depsi: p = 0.001, 136 1 µM: p = 0.1, 136 5 µM: p = 0.1, and 136 10 µM: p = 0.006. For the Hut78 cells (right), Depsi: p = 0.001, 136 1 µM: p = 0.08, 136 5 µM: p = 0.02, and 136 10 µM: p = 0.005. (TIFF) [file pone.0068915.s002.tiff]

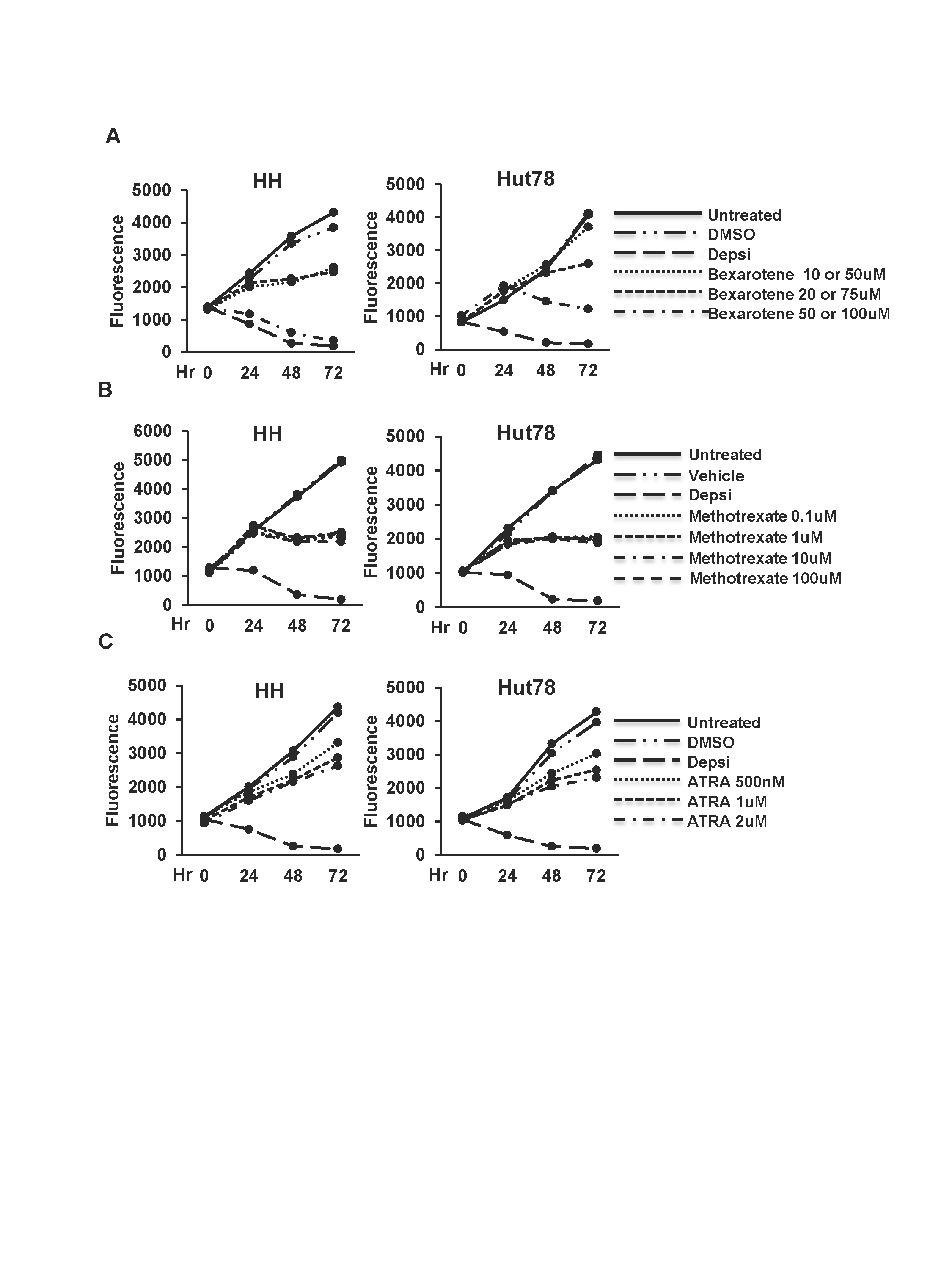

Supplement: Figure S3 — Dose curves for Bexarotene, Methotrexate, and ATRA reveal optimal concentrations for combination treatments. Dose curves of Bexarotene (A), Methotrexate (B), and ATRA (C) treated HH cells or Hut78 cells. Cells were treated at hour 0 with DMSO, 10 nM Depsipeptide (Depsi), or varying concentrations of Bexarotene, Methotrexate, or ATRA. Cell growth was assessed at 0, 24, 48, and 72 hours after treatment. In all studies except for (A), the HH and Hut78 cells were treated with the same varying concentrations of CTCL drugs. HH cells were treated with 10, 20, or 50 µM of Bexarotene while Hut78 cells were treated with 50,75, or 100 µM of Bexarotene. In (B) DMSO and a solution containing Na2CO3 served as vehicle controls. (C) ATRA was administered at hour 0 and re-dosed at 48 hours after treatment. For (A–C), representative curves are shown from experiments performed in triplicate that are consistent with other biological replicates. Statistical analysis was performed using a two-tail paired T-test and comparing the HDI or CTCL drug treated cells to DMSO treated cells resulting in the following p values: (A) HH cells (left), Depsi: p = 0.0007; Bexarotene 10 µM: p = 0.001; Bexarotene 20 µM: p = 0.004; Bexarotene 50 µM: p = 0.001. Hut78 cells (right), Depsi: p = 0.002; Bexarotene 50 µM: p = 0.8; Bexarotene 75 µM: p = 0.1; and Bexarotene 100 µM: p = 0.04. (B) HH cells (left), Depsi: p = 0.001; Methotrexate 0.1 µM: p = 0.007; Methotrexate 1 µM: p = 0.01; Methotrexate 10 µM: p = 0.01; Methotrexate 100 µM: p = 0.006. Hut78 cells (right) Depsi: p = 0.001; Methotrexate 0.1 µM: p = 0.005; Methotrexate 1 µM: p = 0.006; Methotrexate 10 µM: p = 0.004; Methotrexate 100 µM: p = 0.004. (C) HH cells (left), Depsi: p = 0.001; ATRA 500 nM: p = 0.008; ATRA 1 µM: p = 0.002; ATRA 2 µM: p = 0.003. Hut78 cells (right) Depsi: p = 0.001; ATRA 500 nM: p = 0.02; ATRA 1 µM: p = 0.005; ATRA 2 µM: p = 0.006. (TIFF) [file pone.0068915.s003.tiff]

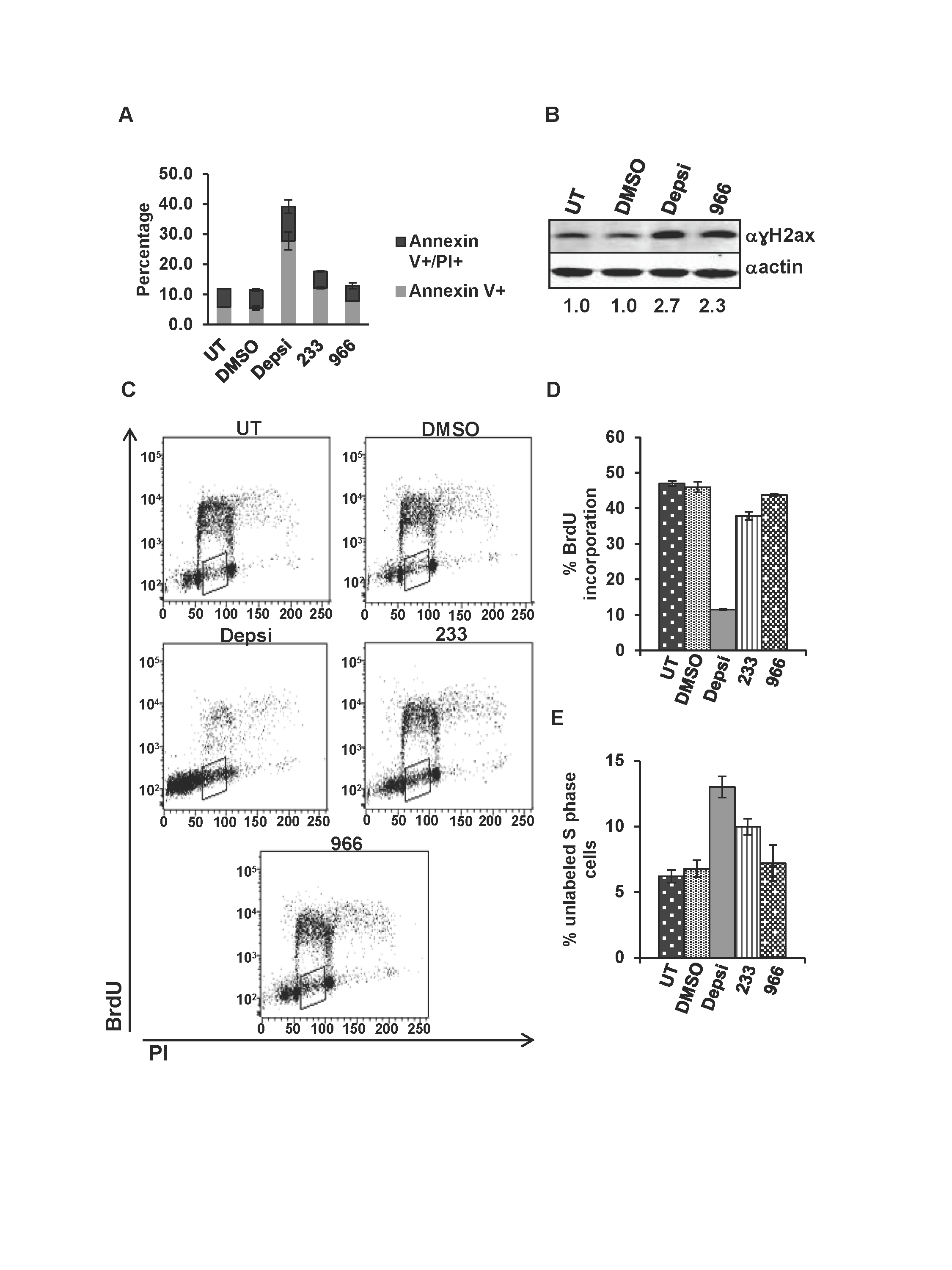

Supplement: Figure S4 — HDIs increased in apoptosis, DNA damage, and cell cycle defects in HH cells. (A) HH cells were treated with DMSO, 10 nM Depsipeptide (Depsi), 10 µM 233, or 10 µM 966 for 24 hr and apoptosis levels were assessed by Annexin V/PI staining and flow cytometry. Untreated (UT) and DMSO treated cells were used as controls. Shown is a representative graph from an experiment performed in duplicate that is consistent with other biological replicates. (B) Western blot analysis of γH2aX levels in HH cells treated with DMSO, 10 nM Depsi, or 10 µM 966 for 8 hrs. Untreated and DMSO treated cells were used as controls. (C) Cell cycle status was analyzed using BrdU/PI and flow cytometry. HH cells were treated with DMSO, 10 nM Depsipeptide (Depsi), 10 µM 233, or 10 µM 966 for 24 hr and pulsed for an hour and a half with BrdU prior to cell harvest and analysis. Shown are representative flow cytometry plots from an experiment performed in duplicate that is consistent with other biological replicates. (D) Graphical representation of BrdU incorporation from the experiment described in (C). (E) Graphical representation of the percent of S phase cells that did not incorporate BrdU (shown by box in panel (C)). Statistical analysis for both the Annexin V and BrdU experiments was performed using a two-tail T-test and comparing the HDI treated cells to the DMSO treated cells resulting in the following p-values: (A) Depsi: p = 0.02, 233: p = 0.01, and 966: p = 0.06. (D) Depsi: p = 0.002, 233: p = 0.05, and 966: p = 0.3. (E) Depsi: p = 0.03, 233: p = 0.07, and 966: p = 0.8. (TIFF) [file pone.0068915.s004.tiff]

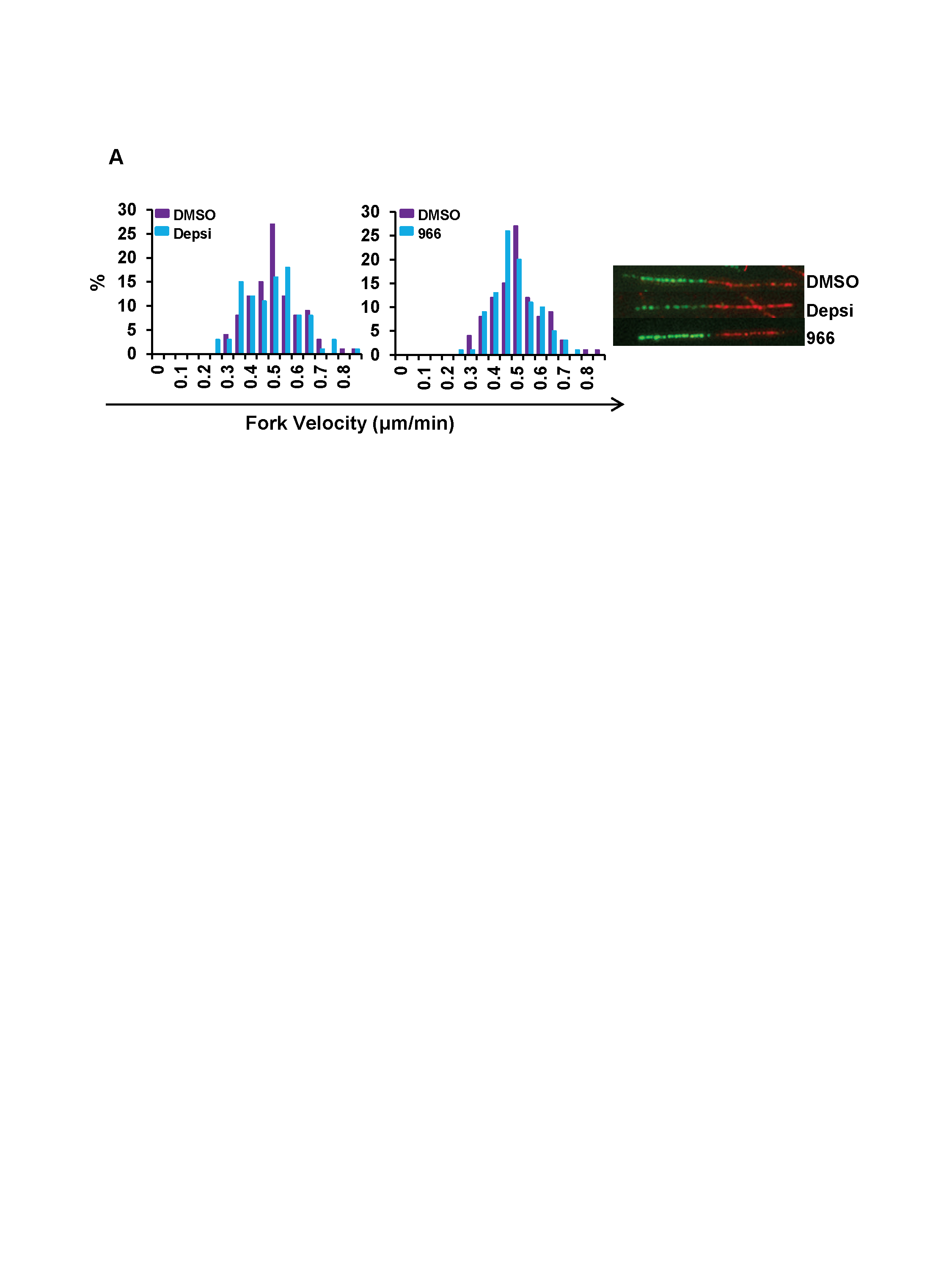

Supplement: Figure S5 — HDI treatment after labeling with IdU and CldU shows no changes in DNA fiber length. (A) DNA fiber labeling analysis was used to assess DNA fiber length in Hut78 cells treated with either DMSO, 10 nM Depsipeptide (left) or 10 µM 966 (right) 4 hrs after labeling the cells with IdU for 20 mins (green) followed by 20 mins of CldU (red). (A) Graphical representation of fork velocity as determined by the total length of fibers (IdU plus CldU) divided by 40 min pulse is shown. Representative measured fibers are shown at the right for DMSO, Depsi, and 966. 100 fibers were measured for each sample. Statistical analysis was performed using Mann-Whitney test and standard deviations were calculated. HDI treated cells were compared to the DMSO treated cells resulting in the following p-values: Depsi: p = 0.5 and 966: p = 0.4. The average velocities for both Depsi and 966 were within 1 standard deviation of the average velocity for DMSO. (TIFF) [file pone.0068915.s005.tiff]
